# Applicability of Raman Spectroscopy for the Assessment of Wheat Flour Quality and Functionality in Bakery Applications

**DOI:** 10.3390/foods14193330

**Published:** 2025-09-25

**Authors:** Justine Van der Vennet, Fien De Witte, Peter Vandenabeele, Mia Eeckhout, Filip Van Bockstaele

**Affiliations:** 1 Research Unit Cereal and Feed Technology, Department of Food Technology, Safety and Health, Faculty of Bioscience Engineering, Ghent University, Valentin Vaerwyckweg 1, 9000 Ghent, Belgium; 2 Food Structure and Function Research Group, Department of Food Technology, Safety and Health, Faculty of Bioscience Engineering, Ghent University, Coupure Links 653, 9000 Ghent, Belgium; 3 Raman Spectroscopy Research Group, Department of Chemistry, Faculty of Sciences, Ghent University, Krijgslaan 281, 9000 Ghent, Belgium

**Keywords:** bakery, Raman spectroscopy, wheat flour

## Abstract

Advancements in Raman spectroscopy have broadened the utilization possibilities for food applications. The present review covers the working principle and methodology of the emerging technique in the context of wheat (flour) as a bakery ingredient. Special attention is paid to the primary constituents of wheat flour, starch and gluten proteins, both in their isolated forms and within complex matrices such as flour, dough, and various end products. This review examines how compositional and structural variations in these components are reflected in their Raman spectra and imaging characteristics and how this can be interpreted in terms of quality and functionality. The review concludes by outlining prospective research directions and future opportunities for advancing Raman-based analysis in cereal and bakery science.

## 1. Introduction

Raman spectroscopy, based on the principles of inelastic light scattering, has evolved into a powerful analytical tool since its discovery by C.V. Raman and K.S. Krishnan in 1928. Due to its non-destructive nature, resistance to water interference, and ability to provide molecular-level insights without extensive sample preparation, Raman spectroscopy has become a valuable asset across numerous disciplines, such as pharmaceutical science, material science, forensic analysis, biology, and food sciences [1,2]. Technological advancements, including excitation through the use of lasers and charge-coupled device (CCD) detectors, have significantly improved its sensitivity and applicability. These developments have refined the methodology and led to the emergence of diverse advanced analytical approaches. Some of these techniques, which are particularly relevant to food science research, are outlined in Section 3 on instrumentation.

In the context of food applications, research has predominantly focused on food safety, with particular emphasis on the detection of adulterants and contaminants, including pathogenic microorganisms, pesticides, food additives, (myco)toxins, etc., which have already been thoroughly reviewed [2,3,4,5]. In contrast, the present review will emphasize the use of Raman spectroscopy to gain molecular-level insights into the primary components of wheat flour, namely starch and gluten proteins, as well as their techno-functional behavior in wheat-flour-based products. This understanding will enable a critical assessment of the technique’s applicability within the bakery sector. Furthermore, its non-destructive nature and rapid measurement capabilities support its potential integration as an in-line monitoring tool. Such implementation aligns with the principles of Process Analytical Technology (PAT), which facilitates real-time monitoring and control of process parameters across various stages of bakery production [6]. This typically involves assessing the wheat (flour) quality, monitoring the mixing and proofing phase, and controlling the baking process. The adoption of PAT in this domain would contribute to enhanced safety, improved product quality, and greater process efficiency [6]. To that end, Raman spectroscopy represents a valuable technique, provided there is a thorough understanding of how Raman signals are influenced by and related to formulation and processing conditions. This review aims to provide an overview of these aspects in the context of bakery products. 

## 2. Working Principle of Raman Spectroscopy

**Figure 1:**
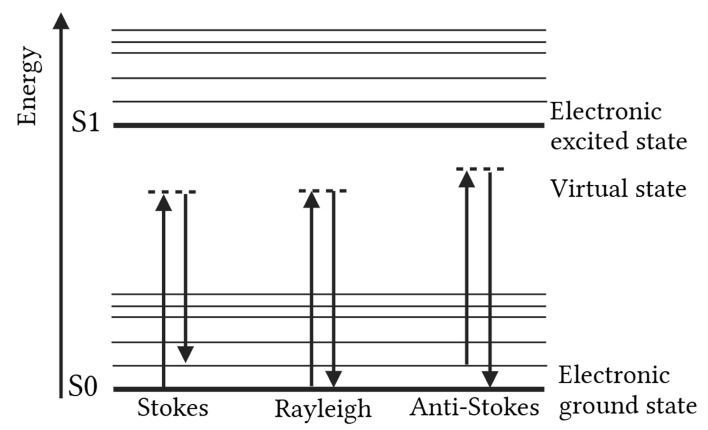
Energy-level transitions (Stokes, Rayleigh, and anti-Stokes scattering) of Raman spectroscopy.

Raman spectroscopy is a vibrational spectroscopic technique, wherein monochromatic light (usually VIS, UV or NIR light) interacts with molecular vibrations, leading to temporary distortion of the electron cloud of the molecule and thereby promoting the system to a higher-energy virtual state. If this distortion leads to a change in molecular polarizability, the vibrational mode is Raman-active. When the molecule relaxes back, a photon is emitted. Most scattered photons undergo elastic Rayleigh scattering, for which the energy and wavelength of the scattered photon are identical to those of the incident photon. However, a small fraction (~0.001%) experiences inelastic scattering, resulting in Stokes and anti-Stokes shifts, which are characterized by, respectively, a lower and higher energy compared to the incoming photons [2,7,8,9,10,11]. Figure 1 schematically visualizes these principles. 

These shifts, expressed in wavenumbers (cm^−1^), along with the intensity of the scattered light, produce a Raman spectrum. This serves as a molecular fingerprint, allowing for the simultaneous identification of various components within a sample. In addition to qualitative analysis, Raman spectroscopy also enables quantification, as the intensity of the scattering is directly proportional to the concentration of the analyte being examined [5,7,8,9]. More detailed information on the working principles of this vibrational technique can be found in Das and Argawal [1] and Vandenabeele [7]. 

## 3. Instrumentation

A basic Raman spectroscopy setup comprises several essential components. These include a laser source for molecular excitation, optical elements for the collection of the resulting Raman-scattered light, a dispersion system to separate the light into its constituent wavelengths, and a detector to capture the Raman signals [7]. While this fundamental architecture remains consistent, a wide range of instrumental configurations have been developed over time. These span from conventional (spontaneous) Raman spectroscopy systems to more sophisticated and specialized variants. The most commonly applied methods in wheat-related research are discussed herein. For a more elaborate overview of different techniques and their broader food applications, the reader is referred to Das and Argawal [1], Fakayode et al. [8], and Wu et al. [2].

One of the most straightforward modifications of Raman instrumentation involves the selection of the laser excitation wavelength. Commonly used lasers in the wheat-based research include the 785 nm diode laser and the 1064 nm Nd:YAG laser, where the latter is frequently employed in Fourier-transform (FT) Raman spectroscopy, alongside other widely used wavelengths, such as 633, 532, and 473 nm [7,11]. Longer-wavelength lasers, which emit lower-energy photons, are particularly advantageous for biological samples like wheat and wheat-based products. This is primarily due to the reduced risk of sample degradation or burning, and the lower propensity to induce fluorescence, which can otherwise severely overwhelm the Raman signals. This benefit results in an improved signal-to-noise ratio, enabling the detection of subtle spectral features [12]. However, the shift to longer wavelengths comes at the expense of weaker Raman scattering and thus lower signal intensities [7,11,13]. Besides the longer wavelength excitation source, FT-Raman differs from a dispersive system by using a (Michelson) interferometer instead of a diffraction grating. This interferometer, consisting of a beam splitter and a stationary and a movable mirror, introduces an optical path difference between two split beams, producing an interferogram through constructive and destructive interference upon recombination of both beams. This time-domain signal allows for simultaneous collection of the entire spectrum and is converted by means of the mathematical Fourier transformation, yielding the Raman spectrum in the frequency domain [7,11].

**Figure 2:**
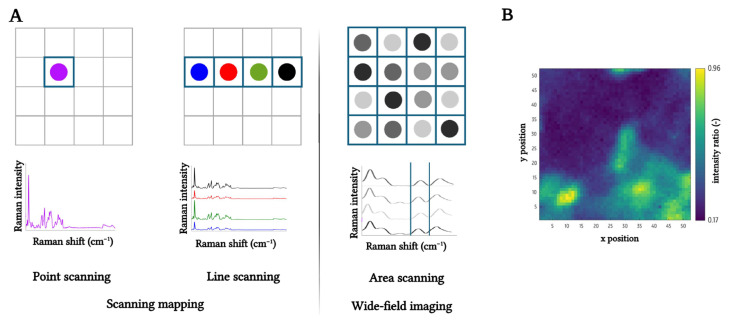
(**A**) Schematic representation of scanning mapping (point and line scanning) and wide-field imaging (area scanning), with the blue zones obtained from one Raman scan, and (**B**) an example of the Raman mapping of rapeseed oil (RPO) oleogel containing 10% carnauba wax (CRW) obtained via point scanning.

Furthermore, to fully harness the potential of Raman spectroscopy, the technique can be supplemented with a microscopic setup to allow for spatial distribution insights (mapping or imaging). A key advantage of this technique is its label-free nature, allowing chemically specific sample analysis without external dyes or labels [14]. Two primary modalities are commonly employed: scanning-based mapping and wide-field imaging. The scanning (mapping) approach often uses a confocal microscope, in which a confocal pinhole eliminates out-of-focus stray light [4,15]. Within scanning-based mapping, two acquisition modes exist, namely point and line scanning. The first approach collects a full Raman spectrum at each spatial coordinate, offering high spectral resolution and coverage of the entire spectral range, but requiring extensive acquisition time and posing a risk of laser-induced sample damage [4,16]. Alternatively, line scanning uses a line-shaped laser beam to illuminate a broader area simultaneously, significantly accelerating data acquisition [4,17]. In contrast, wide-field Raman imaging captures the entire sample in a single acquisition and uses filters to select the appropriate Raman signal. This enhances efficiency (timing) but limits spectral discrimination, making component differentiation more challenging [4]. A schematic representation of the three aforementioned Raman spatial visualization techniques, together with an illustrative Raman mapping, is presented in Figure 2.

## 4. Wheat Starch

As the main component, starch constitutes approximately 63–72% of wheat flour (based on 14% moisture content), and this polysaccharide is composed of two types of biopolymers: amylose and amylopectin, which account for roughly 25% and 75% of the starch fraction, respectively [18]. However, varying levels between 18.2 and 28.8% have been reported for the amylose content of common wheat, with more extreme levels towards 0 or 38%, and even up to 50–80%, in the case of waxy and high-amylose wheat lines, respectively [19,20]. The latter range for high-amylose wheat lines, however, primarily concerns cultivars developed in North America and Australia. Although both polymers are entirely composed of glucose units, they differ in their structural organization. Amylose is predominantly linear, formed by α-1,4-glycosidic linkages between 1500 and 6000 sugars molecules with relatively few branches. In contrast, amylopectin is highly branched due to the presence of α-1,6-glycosidic bonds in addition to the α-1,4-linkages and a single molecule is significantly larger with 300,000 to 3,000,000 glucose units [18]. Both are present in the form of semi-crystalline granules of generally two different size distributions: the smaller (1–10 µm), spherical, B-type granules and the larger, lens-shaped, type A granules with a diameter ranging from 10 to 35 µm [21]. Internally, these are structured as alternating crystalline and amorphous regions, with amylopectin providing crystallinity and amylose occurring in the amorphous state. The hierarchy starts with single helices forming double helices, which organize into lamellae. These lamellae cluster into blocklets within growth rings, ultimately forming the starch granule [22]. A distinction is made based on whether the packing density of these structures is higher or lower, or if these are randomly organized. They are, respectively, denoted as long-range (crystalline) and short-range ordered structure and amorphous (non-crystalline) regions. 

The functional properties of starch become particularly evident upon heating in the presence of a sufficient amount of water, a process known as starch gelatinization [19]. During this process, starch granules swell, there is loss of crystallinity (i.e., loss of the molecular order of granules) and they eventually become disrupted, leading to the leaching of primarily amylose in the surrounding matrix when exceeding the gelatinization temperature (~52–85 °C) [18]. Upon cooling, retrogradation occurs, which is the reassociation of the starch molecules, adopting a new (semi-)crystalline structure that contributes to the solidification of the baked product. As this reorganization continues throughout storage, it is also responsible for staling, a rather undesirable phenomenon in baked products that shortens the shelf life [18,19]. 

**Figure 3:**
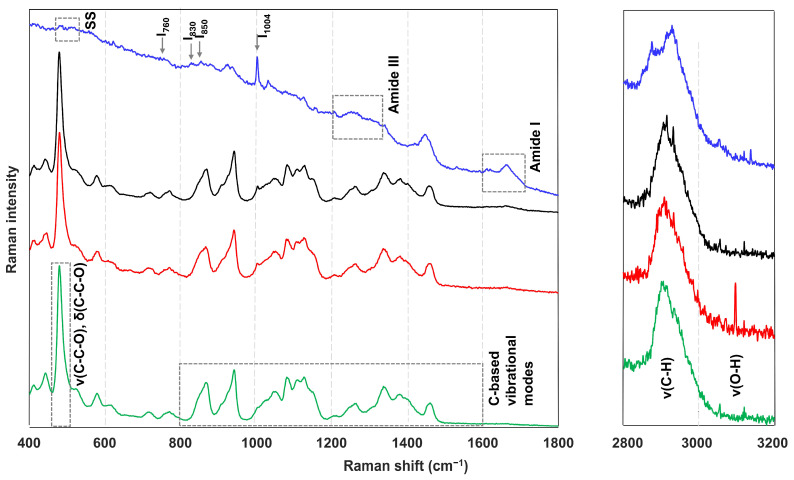
Raman spectra of native wheat starch (green), wheat flour (red), dough (black), and native wheat gluten (blue) in which the band assignments are highlighted (experimental setup in Appendix A.1).

The native starch structure and structural evolutions during heating and cooling have already been extensively studied using various analytical techniques, including X-ray diffraction (XRD), small-angle X-ray scattering (SAXS), differential scanning calorimetry (DSC), Fourier-transform infrared spectroscopy (FTIR), nuclear magnetic resonance (NMR), and Raman spectroscopy [23,24,25,26,27]. XRD provides insights into the long-range crystalline order of starch granules by analyzing the intensities and angles of the diffracted X-rays [22,26], whereas SAXS focuses on variations in the electron density between different zones (amorphous vs. crystalline) at the nanoscale [26]. DSC relies on thermal transitions, specifically the onset (T_o_), peak (T_p_), and conclusion (T_c_) temperatures, to link enthalpic changes to molecular ordering. NMR, FTIR, and Raman spectroscopy are all spectroscopic techniques, and they are considered useful methods to assess the short-range ordered structure [22,26]. In the case of NMR, low-resolution ^1^H NMR is often used, which considers the mobility of starch polymers. This allows for the distinction between the more mobile behavior of gelatinized starch and the more rigid and therefore less mobile retrograded starch [26]. FTIR and Raman spectroscopy are complementary techniques. Both focus on the vibrational modes of molecules: infrared (IR) spectroscopy mainly provides information on asymmetric vibrations in polar groups, while Raman spectroscopy is more suited to detecting symmetric vibrations in nonpolar groups. The resulting spectra can be interpreted in terms of the band positions, intensities, areas, and full width at half maximum (FWHM), providing insights at the molecular level [26]. This is visualized in Figure 3 for native wheat starch, as well as for native gluten, wheat flour and dough, which will be discussed in detail in later subsections. All the single-spectrum measurements were conducted using a confocal Raman microscope equipped with a 785 nm laser. Detailed information regarding the experimental conditions and data processing procedures is provided in Appendix A.1.

To interpret the Raman spectral data of starch, there are recurring regions that draw analytical attention. A prominent and intense Raman band is observed around 480 cm^−1^, as reported by Kizil and Irudayaraj [28] and Mutungi et al. [29]. This band falls within the <800 cm^−1^ region, which is primarily attributed to skeletal ring vibrations of the glucose pyranose unit [22]. Specifically, the 480 cm^−1^ band reflects the degree of polymerization and crystallinity of starch, with a narrower bandwidth (referred to as FWHM) indicating a higher degree of both attributes [22,28,29]. Beyond this region, additional significant Raman bands are found between 800 and 1600 cm^−1^, which are generally associated with carbon-based vibrational modes [13,22,30]. This includes vibrational modes associated with C–O, C–O–H, C–O–C, and CH_2_ groups [13,22,30]. Additionally, the spectral regions between 2800–3000 cm^−1^ and 3000–3600 cm^−1^ are commonly attributed to C–H stretching and O–H stretching modes, respectively [22,28]. All the aforementioned bands and their corresponding assignments are listed in Table 1. 

To gain insight into the starch structural changes induced by key processing steps, studies commonly focus on alterations in the aforementioned Raman bands. The exact methodological setups, including the instrumentation, parameters, and applied (pre-)processing methods that were used in research on isolated wheat starch, are summarized in Table 2. 

In the case of gelatinization, particular attention is paid to changes in the short-range, local molecular order, an area for which Raman spectroscopy is particularly well-suited. Heating disrupts the long-range order while retaining some short-range structure, thereby creating a so-called more amorphous state [31]. Research efforts frequently target the characteristic 480 cm^−1^ Raman band, measured in starch–water mixtures, assessing its intensity [28,31,32,33,34], area [31,33,34], and FWHM [31,32] to link with the molecular ordering changes. It has been found that increasing the final heating temperature when applying a gradually increasing heating trajectory or higher water contents during heating causes a greater decrease in the intensity and area of this band and is thus interpretative for less short-range order [31,32,33]. The FWHM of the 480 cm^−1^ band is proposed to be a reliable indicator of the short-range molecular order in (crystalline) starch [22,31,32,34]. Wang et al. [32] reported that increasing the final heating temperature results in higher FWHM values, indicating a greater loss of short-range order. In contrast, the study by Liu et al. [31], which examined the effect of varying the water content on amorphous wheat starch, did not observe significant changes in this parameter. In addition to the 480 cm^−1^ band, Liu et al. [31] also analyzed several other bands, and among these, the bands at 1080 cm^−1^ (associated with C–OH bending) and 1131 cm^−1^ (C–O stretching) were likewise identified as useful indicators of the short-range molecular order due to their overall high intensities. Building on these findings, the same group used Raman spectroscopy for the quantification of the short-range order in non-crystalline starch [34]. This was achieved by calculating the area of the corrected spectrum of gelatinized starch (i.e., the spectrum after subtracting that of an amorphous reference) relative to the area of the spectrum of just-gelatinized starch.

Gelatinization is consistently followed by retrogradation, a phenomenon that has been extensively studied. In this context, the influence of the final heating temperature and water content [33,35,36], the addition of fatty acids [37], and the storage time [36,38,39] on retrogradation behavior have all been examined through analysis of the characteristic 480 cm^−1^ Raman band, particularly in terms of the FWHM and position shift. In the study by Huang et al. [33], the FWHM was measured after 7 days of storage at 4 °C. When comparisons were made at the same final heating temperature, a decrease in the FWHM was observed at intermediate water contents (42.9% and 50.0%), while a broadening occurred at the highest applied water content of 60%, with the latter indicating reduced molecular order. A similar trend in the FWHM with varying water content was not observed in the study by Wang et al. [35]. However, these authors did report a clear difference between gelatinized starch (FWHM_G_) and retrograded wheat starch that had been reheated after 7 days of storage at 4 °C (FWHM_R_), with higher values of FWHM_R_ interpreted as lower relative crystallinity in the retrograded samples. In contrast, Wang et al. [37] focused on the effect of fatty acids on normal wheat starch (NWS) and waxy wheat starch (WWS), given the well-known retrogradation-delaying effect of lipids. Again, the FWHM of the 480 cm^−1^ band was analyzed. While WWS showed no significant changes in the FWHM, suggesting minimal amylopectin–lipid complexation, the NWS samples displayed a consistent decrease in the FWHM with decreasing fatty acid chain length (NWS > NWS–Palmitic acid > NWS–Myristic acid > NWS–Lauric acid), indicating enhanced complex formation and a corresponding reduction in retrogradation. Monitoring retrogradation over time, as performed by Fechner et al. [38] via the position shift of the same characteristic band over 24 h, showed that the shift was limited in wheat starch compared to potato and maize starch. Liu et al. [36] and An et al. [39] included longer storage durations at 4 °C: up to 21 and 35 days, respectively. These studies showed a progressive decrease in the FWHM at 480 cm^−1^, pointing to increasing short-range molecular order during continued retrogradation. An et al. [39] further observed that the FWHM decrease was more pronounced between days 0 and 7 than between days 7 and 35. Additionally, Liu et al. [36] examined the impact of varying the water content. Across all the storage periods, an increase in water content during gelatinization resulted in broader bands, indicating a reduction in the residual short-range molecular order that is left after gelatinization, which in turn limits the extent of retrogradation. An et al. [39] also obtained further insights from FWHM analysis of the bands at 856 and 1343 cm^−1^, which are associated with C(1)–H, CH_2_, and α-1,4-glycosidic bond (C–O–C) skeletal vibrations, respectively. These bands exhibited similar trends to the 480 cm^−1^ band. In addition, two-dimensional correlation spectroscopy (2D-COS) was used to track the changes in important bands over time via color plots, indicating whether the bands showed similar behavior (e.g., all increasing or decreasing) and the sequential order in which the changes occurred. 

**Table 2:** Methodological setup of research conducted on (isolated) wheat starch, as outlined in the corresponding article.

Sample Type	Raman Device	Laser Wavelength (nm) and Power (mW)	Resolution (cm^−1^)	Number of Scans/Accumulations	Raman Shift Range (cm^−1^)	(Pre-)Processing Steps	Reference
**Starch/water suspensions**	FT-Raman	1064 3000	16	256	400–4000	Area normalization, baseline correction	[28]
**Starch/water mixtures**	LCM ^1^-Raman	785 N.S. ^2^	7	8	100–3200	N.S.	[31]
**Starch/water systems**	LCM-Raman	785 N.S.	N.S.	5	100–3200	N.S.	[32]
**Starch/water mixtures**	LCM-Raman	785 N.S.	7	N.S.	100–3200	N.S.	[33]
**Starch/water mixtures**	LCM-Raman	785 N.S.	7	N.S.	100–3200	Baseline correction	[34]
**Starch/water mixtures**	LCM-Raman	785 N.S.	N.S.	5	100–3200	N.S.	[35]
**Starch/** **fatty acid mixtures**	LCM-Raman	785 N.S.	7	N.S.	100–3200	N.S.	[37]
**Starch/water mixtures**	FT-Raman	1064 250	4	400	N.S.	Blackman–Harris 4 apodization	[38]
**Starch/water mixtures**	Portable Raman	785 300,000	N.S.	3	200–2000	Calibration, baseline correction	[39]

^1^ Laser confocal micro-Raman (employed for single-point measurements). ^2^ N.S. means ‘Not specified’.

## 5. Gluten Proteins

In addition to starch, proteins—and particularly gluten proteins—play a crucial role in wheat-based products. The protein content of commercial wheat flour typically ranges from 7% to 15% (based on 14% moisture content), and this parameter serves as a key criterion when determining flour’s suitability for specific applications [18,40]. Wheat proteins can be classified, according to the Osborne classification, into water-soluble albumin and globulin (soluble in salt solution), accounting for ~15% of the total protein content, and gluten-forming proteins, which constitute the remaining 85%. The latter group is further subdivided into gliadins, which are soluble in aqueous alcohol, and glutenins, which are soluble in dilute acids and bases [18]. Gliadins are considered globular monomeric proteins and have molecular weights ranging from 30 to 75 kDa [41]. Among the gliadin subtypes, α- and γ-gliadins contain intramolecular disulfide bonds formed by cysteine residues (mostly located at the C-terminal end of the primary structure), while ω-gliadins lack cysteine. Structurally, gliadins feature β-turns in the N-terminal domain and a combination of α-helices and β-sheets in the C-terminal domain. In contrast, glutenins are polymeric proteins with much higher molecular weights, often exceeding several tens of millions of kilodaltons. They are composed of high-molecular-weight (HMW) and low-molecular-weight (LMW) subunits, which are interconnected via intermolecular disulfide bonds. The presence of cysteine, especially in the N- and C-terminal domains of these subunits, is crucial for the formation of these crosslinks. In these regions, α-helices are a common secondary structure feature. Two other amino acids play particularly important roles in the structure and functionality of gluten proteins: glutamine and proline. Glutamine is abundantly present in both gliadins and glutenins, particularly in the central repetitive domains, and contributes significantly to hydrogen bonding. Proline, a cyclic amino acid frequently occurring in these domains, disrupts the formation of regular secondary structures such as α-helices and β-sheets, thereby imparting flexibility to the protein conformation. 

These gluten-forming proteins are of particular interest from a functional perspective. They are responsible for the formation of a three-dimensional, continuous gluten network during dough mixing in the presence of water. This is required to enable gas retention within the dough and thereby allow the leavening process to occur. This network’s viscoelastic behavior arises from the combined contributions of gliadins, which impart viscous properties and thus extensibility, and glutenins, which confer elasticity [41]. Upon heating, the loaf volume and textural characteristics are established through solidification of the gluten network. Heating induces glutenin polymerization in the temperature trajectory between 55 and 75 °C, followed by glutenin–gliadin crosslinking above 90 °C [42]. These polymerization reactions are fundamentally driven by the crosslinking of thiol groups (-SH) through oxidation to form covalent disulfide bonds (S–S), in addition to thiol–disulfide exchange reactions and non-covalent interactions. 

A range of analytical techniques are available to investigate the structure of (gluten) proteins. Kłosok et al. [43] categorize these techniques into indirect and direct methods. Indirect methods provide information related to aggregate formation, for which techniques such as electrophoresis, size-exclusion high-performance liquid chromatography (SE-HPLC), reversed-phase HPLC (RP-HPLC), and liquid chromatography–mass spectrometry (LC-MS) are commonly used. Thermogravimetric methods and DSC, as indirect methods, reveal changes in thermal properties, whereas NMR is used to assess alterations in water population and hydrogen bonding behavior. The category of direct methods includes XRD, circular dichroism (CD), infrared (IR), and Raman spectroscopy. The latter three are spectroscopic techniques primarily focused on elucidating protein secondary and tertiary structures. XRD enables the investigation of higher-order three-dimensional structural organization. Circular dichroism uses polarized light, of which the signals after interacting with (gluten) proteins can be linked to specific secondary structural characteristics such as α-helices, β-sheets, etc. FTIR, Raman spectroscopy, and XRD were previously discussed in the context of starch analysis; the same underlying working principles apply to protein structural characterization. For a more comprehensive overview of the available analytical techniques, the reader is referred to Lan et al. [44].

Given the importance of gluten in determining both dough behavior and final product characteristics, the impact of processing, and particularly the addition of ingredients, on its structural and molecular conformations (namely, secondary and tertiary structures) has been widely investigated with Raman spectroscopy. In this context, several recurring spectral regions of interest have been identified and are visualized in Figure 3. One such region is the disulfide bond (S–S) region (470–550 cm^−1^), which consists of three stretching vibrational bands at approximately 470–515 cm^−1^, 515–525 cm^−1^, and 535–545 cm^−1^. These sub-regions correspond to different disulfide bond conformations: gauche–gauche–gauche (g–g–g), gauche–gauche–trans (g–g–t) (also referred to as trans–gauche–gauche (t–g–g)), and trans–gauche–trans (t–g–t), respectively [45]. Among these, the g–g–g conformation is considered the most stable. To resolve the overlapping signals in this region, derivative processing and subsequent deconvolution are often applied as pre-processing techniques [46]. Mixed Lorentzian–Gaussian line-shape fitting can be applied to extract individual band positions in order to assign them to one of the three aforementioned conformations and to assess whether the position is shifted upon treatment [45]. Furthermore, the percentage distribution of the conformations is achieved by area calculation under the fitted curve. In addition, several aromatic amino-acid-associated bands are of interest. They appear at 760 cm^−1^, 830 and 850 cm^−1^, and 1004 cm^−1^ and correspond to tryptophan, tyrosine, and phenylalanine, respectively. The intensity of the tryptophan band at 760 cm^−1^ is sensitive to the hydrophobicity of the indole ring’s microenvironment; higher intensities are typically indicative of a buried indole ring, which may suggest a more ordered or folded protein structure [45]. The tyrosine-associated bands at 830 and 850 cm^−1^ are often expressed as a ratio, known as the tyrosine doublet ratio (I_850_/I_830_), which provides insights into the local environment of tyrosine residues. An increased tyrosine ratio is generally associated with greater exposure of tyrosine residues [45]. A lower intensity at 850 cm^−1^ compared to I_830_ is interpreted as a more buried state, meaning involvement in intermolecular and intramolecular interactions [47]. Ratio values within the range of 0.90 to 1.43 are typically interpreted as tyrosine, more specifically its phenolic hydroxyl group, acting as both a hydrogen bond donor and acceptor, whereas higher ratios suggest a predominant role as a positive charge acceptor [47,48]. Another region of importance is the amide I band, typically ranging from ~1600 to 1700 cm^−1^ and related to the C=O stretching vibrations of the amide group [49]. This region provides key information about the protein secondary structure, with characteristic sub-bands assigned to aggregates, β-sheets (hydrogen bonded or not), solvated helices, random coils, α-helices, β-turns (hydrogen bonded or not), and antiparallel β-sheets (hydrogen bonded or not) [45,50,51]. The exact positions of these sub-bands can vary slightly depending on the literature source (see Table 3, in which all the bands and their corresponding assignments are listed). Position shifts are again considered, next to the calculation of the percentage distributions of the different conformations based on areas under the curve-fitted band profiles (i.e., deconvoluted bands) achieved by the previously mentioned derivative processing and deconvolution [46]. Additionally, though less frequently considered due to the typically lower signal intensity, the amide III region (ranged between 1200 and 1330 cm^−1^ and related to C–N stretching and N–H bending), also offers insights into the protein secondary structures [49,50]. Though rarely a focus, the C–H stretching region (2800–3100 cm^−1^) is associated with hydrophobic groups [45], while the region 3100–3400 cm^−1^, presenting OH group vibrations, can include contributions from hydrogen bond vibrations [52]. All of the following studies using these Raman characteristics have their corresponding methodological setup listed in Table 4.

**Table 3:** Band assignments for Raman spectral data of wheat gluten.

Raman (cm^−1^)	Band Assignment—Abbreviation	Reference
**450–550; 500–560; 490–545**	**SS bonds**	[45,50,51]
470–515; 500; 502–512	SSg–g–g	[45,50,51]
515–525; 510–520; 514–526	SSg–g–t or SSt–g–g	[45,50,51]
535–545; 540–545; 531–539	SSt–g–t	[45,50,51]
760	Tryptophan—Trp	[45]
1340; 1360	Tryptophan doublet (I_360_/I_340_)	[50]
830; 850	Tyrosine—Tyr Tyrosine doublet (I_850_/I_830_)	[45,50,51]
1004	Phenylalanine—Phe	[45]
**1200**–**1330**	**Amide III**	[50]
1220–1250	β-sheet—βS	[50]
1250–1270	Random coil—RC	[50]
1270–1295	β-turn—βT	[50]
1295–1330	α-helix—αH	[50]
**1600**–**1700; 1570–1720**	**Amide I**	[45,46,50,51]
1604; 1594–1604	Aggregates—AGR	[46,51]
1607–1609	Hydrated β-sheet—hβS	[51]
1615–1625	Pseudo-β-sheet—pβS	[51]
1613–1625; 1630–1640; 1630–1636	β-sheet—βS	[45,50,51]
1625–1637	Solvated helix	[45]
1637–1645; 1640–1650	Random coil—RC	[45,50]
1639–1665	Hydrogen-bonded β-turn—HbβT	[51]
1650–1658; 1648–1658	α-helix—αH	[45,50,51]
1666–1673; 1670–1678; 1667–1675	β-turn—βT	[45,50,51]
1677–1683	Hydrogen-bonded antiparallel β-sheet—HbaβS	[51]
1675–1695; 1690–1700; 1691–1696	Antiparallel β-sheet—aβS	[45,50,51]
2800–3000	CH stretching—ν ^1^(C–H)	[45]
3100–3400	OH stretching—ν(O–H)	[52]

^1^ ν: mode involves a change of bond length (stretching).

**Table 4:** Methodological setup of research conducted on (isolated) wheat gluten, as outlined in the corresponding article.

Sample Type	Raman Device	Laser Wavelength (nm) and Power (mW)	Resolution (cm^−1^)	Number of Scans/Accumulations	Raman Shift Range (cm^−1^)	(Pre-)Processing Steps	Reference
**(Thermal-treated) gluten/WEAX ^1^**	FT-Raman	N.S. ^2^	N.S.	N.S.	N.S.	Baseline correction, normalization	[42]
**Washed-out gluten/SSL ^3^** **/DATEM ^4^** **/SSL + DATEM**	FT-Raman	1064 500	6	1000	N.S.	Baseline correction, normalization, deconvolution	[45]
**Washed-out gluten/dietary fibers**	FT-Raman	1064 1000	8	256	150–3500	Baseline correction, normalization, deconvolution, difference spectra	[46]
**(Thermal-treated) gluten/WEAX**	FT-Raman	1064 251	4	256	50–3500	Baseline correction, normalization	[47]
**Kernel endosperm, gliadin**	FT-Raman	1064 500 and 800	N.S.	500 and 2000	300–200 and 300–4000	Normalization, deconvolution	[48]
**Washed-out gluten/phenolic acids**	FT-Raman	1064 1000	8	256	150–3500	Baseline correction, normalization, difference spectra	[51]
**Washed-out gluten/phenolic acids**	FT-Raman	1064 1000	8	256	150–3500	Baseline correction, normalization, difference spectra	[53]
**(Thermal-treated) gluten, glutenin, gliadin**	Raman imaging spectrometer	785 15	4.7–8.7	2	400–3000	Baseline correction, smoothing, normalization	[54]
**(Thermal-treated) fresh and frozen-stored gluten**	FT-Raman	1064 N.S.	4	800	400–4000	Baseline correction, normalization	[55]
**Thermal-treated gluten, glutenin, gliadin**	Confocal Raman spectrometer	1064 N.S.	1	N.S.	400–3500	Baseline correction, normalization, deconvolution	[56]
**Gliadin/salt/salt + Q ^5^**	Raman microscope	785 N.S.	N.S.	N.S.	500–4000	Baseline correction, smoothing, normalization, standardization	[57]
**Gluten/salt**	Microscopic Raman imaging spectrometer	785 35	4.7–8.7	3	N.S.	Baseline correction, normalization, deconvolution	[58]
**Washed-out gluten/salts**	FT-Raman	N.S.	N.S.	N.S.	400–4000	Baseline correction, normalization	[59]
**Gliadin/glutenin/gluten/salt/salt + CMC ^6^**	Raman spectrometer	785	N.S.	N.S.	N.S.	Baseline correction, normalization	[60]
**Washed-out gluten/SSL**	FT-Raman	1064 500	6	1000	N.S.	Baseline correction, normalization, deconvolution	[61]
**Extracted gliadin/anthocyanins**	FT-Raman	1064 60	4	15000	N.S.	Baseline correction, deconvolution	[62]
**Washed-out gluten/phenolic acids**	FT-Raman	1064 1000	8	256	N.S.	Baseline correction, normalization, difference spectra	[63]
**Washed-out gluten/phenolic acids**	FT-Raman	1064 1000	8	256	150–3500	Baseline correction, normalization, difference spectra	[64]
**Washed out gluten/phenolic acids**	Raman microscope	1064 N.S.	8	256	500–4000	Baseline correction, normalization, difference spectra	[65]
**Washed-out gluten/flavonoids/glycosides**	FT-Raman	1064 1000	8	256	150–3500	Baseline correction, normalization, deconvolution, difference spectra	[66]
**Extracted gliadin/flavonoids/glycosides**	FT-Raman	1064 1000	8	256	150–3500	Baseline correction, normalization, deconvolution, difference spectra	[67]
**Washed-out gluten/hydro-colloids**	FT-Raman	1064 500	6	1000	500–4000	Baseline correction, normalization	[68]
**Washed-out gluten/dietary fibers**	FT-Raman	1064 1000	8	256	150–3500	Baseline correction, deconvolution, difference spectra	[69]
**Washed-out gluten/dietary fibers**	FT-Raman	1064 1000	N.S.	N.S.	150–3500	Baseline correction, normalization, deconvolution, difference spectra	[70]
**Washed-out gluten/dietary fibers**	FT-Raman	1064 1000	8	256	150–3500	Baseline correction, normalization, deconvolution, difference spectra	[71]
**Washed-out gluten/dietary fibers**	FT-Raman	1064 1000	8	256	150–3500	Baseline correction, normalization, deconvolution, difference spectra	[72]
**Washed-out gluten/dietary fibers**	FT-Raman	1064 1000	8	256	150–3500	Baseline correction, normalization, deconvolution, difference spectra	[73]
**Washed-out gluten/moisturized dietary fibers/non-moisturized dietary fibers**	FT-Raman	1064 1000	8	256	150–3500	Baseline correction, normalization, deconvolution, difference spectra	[74]
**Washed-out gluten/oil pomaces**	FT-Raman	1064 1000	8	256	150–3500	Baseline correction, normalization, deconvolution, difference spectra	[75]
**Gluten/wheat bran dietary fiber**	FT-Raman	532 10	0.6	32	400–4000	N.S.	[76]
**Washed-out gluten/KGM ^7^**	FT-Raman	1064 370	4	256	100–3700	Baseline correction, normalization	[77]

^1^ Water-extractable arabinoxylan. ^2^ Not specified. ^3^ Sodium stearoyl lactylate. ^4^ Diacetyl tartaric acid esters of monoglycerides. ^5^ Quercetin. ^6^ Carboxymethylcellulose. ^7^ Konjac glucomannan.

A specific focus was placed on gliadins, which were examined both in halved wheat kernels and as isolated fractions from a normal and an ω-gliadin-deficient wheat line [48]. In the kernel endosperm, the normal wheat line predominantly exhibited the SSt–g–g conformation, while in the absence of ω-gliadins, a higher proportion of SSt–g–t was observed. Furthermore, both lines showed a high tyrosine doublet ratio, indicating that tyrosine residues were exposed. The 760 cm^−1^ tryptophan band displayed lower intensity in the deficient line, suggesting a more exposed state of tryptophan in this genotype. In the isolated gliadin fractions, the amide I region was specifically analyzed, revealing that the ω-gliadin-deficient line contained a higher proportion of β-structures (sheets and turns) at the expense of α-helices and random coils.

The most critical processing step influencing the gluten network development, namely the kneading phase, was examined as part of the study by Krekora et al. [53]. They also investigated the impact of phenolic components, thereby only focusing on the amide I region when it concerned the untreated, washed gluten extracted from a model dough composed of starch and gluten in an 80:15 (*w*/*w*) ratio kneaded for 30 or 60 min. Extended kneading (60 min) increased the presence of aggregates, β-sheets, and random coils, indicating partial disruption of the viscoelastic network, while 30 min favored β-turns and antiparallel β-sheets. Given the predominance of β-structures in glutenins, these types of proteins are considered key contributors to gluten network formation. 

Another key processing step is thermal treatment, which was systematically investigated by Xu and Kuang [54], who studied gluten, as well as glutenin and gliadin when, respectively, different heating temperatures (ranging between 25 °C and 95 °C) were applied. In all three sample types over all the different temperatures, the g–g–g disulfide bridge conformation was the most prevalent. In gluten specifically, a rising trend in both this conformation and the t–g–g conformation was observed with increasing temperature, suggesting aggregation behavior between glutenin molecules and between glutenin and gliadin during heating. The association between glutenin and gliadin with increasing temperature was further supported by two observations: the significantly higher increase in the I_760_ (intensity at the 760 cm^−1^ band) in gluten compared to the isolated proteins, indicating more buried tryptophan residues; and the reduction in the tyrosine ratio. The latter also provided insight into the thermal sensitivity of hydrogen bonding, as the lowest value was found at 75 °C. In terms of the secondary structure, native gluten, glutenin, and gliadin have high percentages of α-helices. The first conformation decreased upon heating by 19.26%, 20.23%, and 21.88%, respectively. Conversely, β-turns increased progressively with temperature and were generally the dominant structure in glutenin and gliadin. The results clearly indicate that α-helix and β-sheet structures are converted into β-turns and random coils as hydrogen bonding weakens and disrupts under heat. These observations regarding the disulfide bridge conformations, and the tryptophan and tyrosine Raman band characteristics at various heating temperatures, align with the findings about gluten reported in the studies of Zhu et al. [47], Wang et al. [42], and Wang et al. [55]. Notably, Zhu et al. [47] reported no change in I_760_, with decreases in SSg–g–g upon heating. Wang et al. [42] observed no significant changes in the aromatic amino acid bands at 95 °C compared to control gluten at 25 °C. Similarly, Wang et al. [55] found no significant difference in I_755_ and no change in the SSt–g–g content at 95 °C relative to the control. The effect of the cooking time on the three aforementioned sample types was investigated in the study by Liu et al. [56]. In the native state, the g–g–g conformation was most abundant in the gliadin fraction, whereas gluten and glutenin predominantly exhibited the t–g–t conformation. Upon prolonged heating for 15 min, the proportion of g–g–g conformation increased for gluten and glutenin, while remaining unchanged for gliadin. Conversely, the t–g–t content decreased in gluten and glutenin but increased in gliadin. These changes suggest a progressive solidification of the tertiary structure involving disulfide bonds in gluten and glutenin, whereas the consistently high prevalence of the stable g–g–g conformation in gliadin may contribute to its greater thermal resistance. Distinct behavior was also observed in the tryptophan Raman band between gliadin, which did not change, and gluten and glutenin. The latter group exhibited a slightly decreased intensity after 5 min of cooking, followed by an increase after 15 min, which is indicative of reaggregation of gluten proteins, followed by network deterioration. Additionally, the tyrosine doublet ratio increased overall, with a noticeable rise already after 5 min of cooking, suggesting that early thermal exposure is primarily responsible for hydrogen bond disruption and unfolding.

In addition to the hydrothermal treatment of fresh gluten, Wang et al. [55] investigated the effects of hydrothermal processing combined with frozen storage. Their findings indicated that frozen storage of control samples at 25 °C led to an increased presence of the g–g–g conformational state, with no significant difference observed between storage durations of 30 and 60 days. This conformational shift was accompanied by a corresponding decrease in the t–g–t state. However, upon heating to 95 °C, both frozen-stored samples showed a complete absence of SSg–g–g and a significant increase in t–g–t content, in contrast to fresh gluten heated to the same temperature. At treatment temperatures of 50 °C and 70 °C, the Raman intensity around 755 cm^−1^ peaked for both fresh and frozen-stored gluten, indicating unfolding, but decreased at 95 °C across all three sample types, suggesting crosslinking and polymerization. Upon application of heating, the tyrosine ratio (I_854_/I_830_) decreased, but overall, it remained within the range of 1.2–1.75 for both fresh and frozen samples, indicating the exposure of tyrosine residues and thus reduced hydrogen bonding strength. 

Furthermore, considerable attention has been directed toward the conformational changes that (isolated) gluten and gliadin proteins undergo upon interaction with various components. These include salt(s) [57,58,59,60] and emulsifiers [45,61], both showcasing improved dough handling due to their interaction with gluten proteins, and phenolic compounds [51,53,57,62,63,64,65,66,67] and dietary-fiber-related compounds [46,47,68,69,70,71,72,73,74,75,76,77], which are incorporated from a health point of view but can show interfering effects on the gluten network. 

Salt addition is a common practice in dough preparation, as it significantly influences dough elasticity and extensibility, which are properties primarily governed by the gluten network [59]. This topic was the main focus of the study by Wang et al. [57] on wheat gliadin and was also addressed in investigations by Kuang and Xu [58] and Yang et al. [59] on gluten. Tang et al. [60] even considered all three fractions: gliadin, glutenin and gluten. However, contradictory effects have been reported concerning the disulfide bond conformations upon salt interaction. For instance, Wang et al. [57] and Tang et al. [60] observed an increase in the t–g–t conformation and a corresponding decrease in the g–g–g conformation across all the tested salt concentrations. The latter conformation was for all protein fractions most prevalent in the control sample. This was interpreted by Wang et al. [57] as salt-induced disruption of interchain disulfide bonds within gliadins. In contrast, both Kuang and Xu [58] and Yang et al. [59] investigated control gluten for which SSg–g–g was not the dominant form and reported an increase in this conformation, alongside a reduction in t–g–t structures. These latter findings suggested that salt may promote enhanced structural stability of these gluten [58]. Regarding aromatic amino acids, increased intensity at the tryptophan band was observed for all the fractions in the study by Tang et al. [60] and the gluten in the study of Kuang and Xu [58], whereas the gliadin of Wang et al. [57] showed a decrease in the tryptophan band intensities at the typical 760 cm^−1^ band and at 1340 cm^−1^ (except that, for the latter band, the intensity increased again to a level comparable to that of salt-free gluten at high NaCl concentration). Moreover, differing effects have also been reported for the tyrosine-associated bands upon salt addition, namely an increased [57,58,60] and decreased [60] ratio, thereby dealing with other fractions in the presence of differing salt concentrations (10 to 200 mmol L^−1^ in case of Wang et al. [57], and 1–3% and 1–4% on gluten weight for Kuang and Xu [58] and Tang et al. [60], respectively). 

Emulsifiers, such as sodium stearoyl lactylate (SSL) [45,61] and diacetyl tartaric acid esters of monoglycerides (DATEM) [45], are frequently added to improve bread quality. In the disulfide bond region, additional bands appear under the application of the additives that are characteristic of t–g–g conformations, in addition to the already present g–g–g band observed in control gluten, though this band shows a peak shift upon treatment. Regarding tryptophan (measured at a different position, namely 875 cm^−1^, in the study by Ferrer et al. [61]), both studies reported increased intensity in treated gluten samples. The tyrosine doublet generally showed a decrease in intensity upon emulsifier addition, with the exception of the highest applied concentration of SSL, which caused an increase. This may indicate enhanced positive charge acceptor activity, possibly related to an excess of metal ions (Na^+^ and Ca^2+^). The amide I region showed that α-helices are the most prevalent secondary structures, with a higher proportion observed when applying an emulsifier. This increase is accompanied by a decrease in β-sheet and random coil structures and, together with the changes in the microenvironment of tryptophan and tyrosine, suggests folding and stabilization of the gluten structure. This is in accordance with their reported mechanism of action as the emulsifying agents would bind to hydrophobic sites in gluten, thereby promoting protein aggregation [45,61]. Additional information was obtained from the CH stretching region (2800–3100 cm^−1^), particularly the 2935 cm^−1^ band, which decreased in relative area upon SSL addition. This decrease is associated with greater burial of tyrosine and hydroxyl-containing groups. The splitting of this band into components at 2939 and 2950 cm^−1^ is linked to amino acid–emulsifier interactions, a phenomenon that was less pronounced with DATEM. 

The impact and interaction of phenolic compounds on the gluten structure, and particularly on gliadins, have been extensively investigated. Owing to their antioxidant properties, these compounds are of growing interest for incorporation into human diets. Among them, phenolic acids and their derivatives have received considerable attention in studies such as those by Krekora et al. [53], Kłosok et al. [51], Krekora and Nawrocka [63,64], and Welc et al. [65], while other studies have focused on flavonoids and related compounds [57,62,66,67]. Incorporation of these types of components results in reversible (hydrogen and hydrophobic bonds and Van der Waals forces) and irreversible (covalent) interactions with gluten, thereby inducing structural and functional changes [65]. Insights into this can be provided with detailed spectral analyses, which primarily focus on the covalent disulfide bond region, bands associated with the microenvironment of aromatic amino acid residues, and the amide I region, of which more detailed changes are presented in Table 5. To this end, model dough systems (80:15 starch-to-gluten ratio) were supplemented with phenolic compounds, from which the gluten or gliadin fraction was isolated. The spectral data of these, and of native gluten or gliadins and pure phenolic compounds, were subtracted and resulted in so-called difference spectra that enable specific gluten or gliadin–phenolic compound interactions to be deduced [51,53,63,64,65,66,67]. Native gluten and gliadin are generally (except for the study by Krekora et al. [67]) dominated by the energetically stable g–g–g disulfide conformation, whereas a varying impact of both phenolic acids and flavonoids were found, ranging from a limited influence and even stabilizing the desired SSg–g–g [51,53,63,65,67] to a decrease in that conformation in favor of t–g–g and/or t–g–t conformations [53,57,62,64,66]. Variations were reported for I_760_ and I_850_/I_830_. Phenolic acid treatments typically resulted in significantly unchanged or increased I_760_ values, while flavonoids showed no consistent trend. The tyrosine doublet generally remained unchanged or showed slight decreases relative to untreated samples, suggesting minimal influence on the local environment of tyrosine residues [53], with the exception of the reported increases in the study of Kłosok et al. [51]. Regarding the amide I region, recurring observations in difference spectra included positive bands for aggregates and β-sheets (both pseudo and hydrated), and negative bands for α-helices and β-turns. These indicate, respectively, a gain and loss of those types of conformations upon interaction with phenolic acids [51,53,63,64,65]. In contrast, such trends were not observed in samples treated with flavonoids or glycosides [57,62,66,67]. The extent to which phenolic compounds modulated all these Raman spectral features, and in turn the gluten structural properties, appeared to depend on several factors: the concentration of the phenolic compound, its molecular structure and functional groups [51,62,64,66,67], its antioxidant activity [53], and its molecular size [64]. These same variables were likewise reflected in supplemented dough farinographic profiles. In these dough consistency–time graphs, the position and magnitude of additional peaks compared to the control sample are related to gluten–phenolic acid interactions, showing an initial strengthening effect that transitions toward mechanical destruction evidenced by reduced consistency in modified doughs [51,53,64].

Another widely discussed group of components are dietary fibers. These substances consist primarily of non-digestible poly- and oligosaccharides, in addition to polyphenols, and are associated with a variety of health-promoting effects. However, their incorporation brings challenges to bread quality, which is inherently linked to the influence they exert on the gluten structure by creating new hydrogen bonds and changing disulfide conformations [46] and by competing with gluten for water [78]. In most studies, a decrease in the stable g–g–g conformation in the disulfide region was observed (exceptions mainly reported by Nawrocka et al. [71,72,73], and Wang et al. [42], for which increases were found), which is typically the dominant structure in native gluten [47,68,69,73,74,75]. The decrease often coincides with an increase in the t–g–t conformation, which is indicative of abnormal protein aggregation and folding [46,47,69,70,71,74]. Principal component analysis in the study by Nawrocka et al. [69] revealed that dough resistance was inversely correlated with g–g–g. Changes in the aromatic amino acid bands were also observed, with a generally decreasing effect on the tyrosine doublet ratio, although still within the range of 0.9 to 1.43, indicating normal tyrosine behavior acting as both hydrogen bond donor and acceptor and thus allowing the formation of inter- and intramolecular hydrogen bonds [42,47,69,70,71,73,76]. No consistent trend was observed for the tryptophan band intensities across the used compounds and concentrations. In the amide I band, a common trend was an increase in antiparallel β-sheet conformation and a decrease in α-helices. Nawrocka et al. [46,70,71,72,73] interpreted this as a dietary fiber component causing α-helices from two protein complexes to connect, resulting in the formation of antiparallel β-sheet structures. Additionally, changes in β-sheet structures that lead to aggregation are linked not only to interactions between side-chain amino acids but also to bonding between amino acid side chains and polysaccharide chains [70,71,73]. However, the precise effects of dietary fiber components largely depend on the specific fibers and the concentration [42,46,68,70,71,72,73,74,75]. Nawrocka et al. [46] indicated that the observed secondary structure changes are mainly linked to the cellulose and pectins present, while anthocyanins and polyphenols occurred in low concentrations and thus have less pronounced effects. Concentration effects were clearly observable in the study of Li et al. [76], in which low (3 to 6%) concentrations of wheat bran dietary fiber resulted in increased hydrophobic interaction with gluten, leading to a more stable, ordered structure, while 9–15% resulted in extensive hydrogen bonds between dietary fiber and protein amide groups, thereby inhibiting gluten crosslinking. The influence of dietary fiber is mainly attributed to water competition between fibers and gluten during dough mixing, thereby leading to partial gluten dehydration [42,46,70,72,73,77]. This effect is evident from the reduced water absorption of gluten in the presence of fibers, as tested by Nawrocka et al. [69], with decreases of up to 25%. This competition is influenced by the solubility and water affinity of the additives [72,73,74]. Insoluble dietary fibers such as cellulose tend to induce more aggregated β-structures, whereas pectins (included in soluble fibers) integrate into the gluten network [74]. Pectins have more hydroxyl groups, and distinctions between citrus and apple pectin effects can be made based on the amount of galacturonic acid, which contains free hydroxyl groups that compete with gluten for available water, thereby compacting the otherwise more flexible hydrated gluten structure [72]. Differences related to the insoluble and soluble dietary fiber contents were also reflected in the farinographic profiles of dough consistency. Fiber addition produced additional peaks, thereby closely resembling the profiles of phenolic-compound-supplemented doughs. Overall, the dehydration and/or gluten modification effects of fibers led to an initial consistency increase, progressing to overmixing [74]. This is in accordance to the increased values for both dough stability and softening index due to Konjac glucomannan addition, as studied by Zhou et al. [77]. In the study by Nawrocka et al. [74], the effect of pre-moisturizing dietary fibers was investigated to reduce gluten dehydration caused by water competition. Washed gluten was analyzed from supplemented model doughs. Limited differences were found in the aromatic amino acid bands between the two groups, suggesting that hydrogen bonding mainly occurred between dietary fiber constituents and water molecules rather than between dietary fiber and gluten proteins. Zhu et al. [47] and Wang et al. [42] studied the effect of heating gluten supplemented with water-extractable arabinoxylan (WEAX). Zhu et al. [47] found that the band corresponding to t–g–t disulfide conformation disappeared at 75 and 95 °C, suggesting that the addition of WEAX contributed to the stabilization of the g–g–g conformation. In contrast, the study by Wang et al. [42] observed more variable effects on disulfide conformations when samples were heated to 70 or 95 °C, with both low- and high-molecular-weight WEAX at different concentrations. Notably, SSt–g–t showed overall increases at both temperatures. Above 70 °C, WEAX caused unchanged [47] and increased [42] tryptophan band intensity and an increased/unchanged [47] and decreased [42] tyrosine ratio compared to untreated gluten at the same temperatures. A more elaborate overview of the impact of dietary fibers on the gluten structure can be found in the review by Zhou et al. [79], which covers Raman studies alongside FTIR, HPLC, and thermal analyses.

## 6. Complex Matrices

Building on the characterization of simplified and isolated systems (wheat starch and gluten proteins), a couple of studies have expanded their focus to more complex food matrices, such as wheat flour, dough systems, and final baked products. The methodological settings of these studies are presented in Table 6. Spectral interpretation in these systems requires careful consideration of the simultaneous presence of multiple constituents. 

One of the initial observations in these more complex systems is the close resemblance between the spectral profile of wheat flour and that of isolated wheat starch, reflecting the predominance of starch within the matrix, while dough and bread spectra similarly resemble that of wheat flour due to its high presence in the formulation, as can be seen in Figure 3 for dough. As noted by Sivam et al. [30], the spectrum of wheat flour effectively represents a combination of individual starch and gluten spectra. Consequently, the same characteristic bands identified in isolated starch and gluten samples are also relevant here. 

Starch-related spectral features remain clearly discernible in wheat flour spectra: the prominent band located at 478 cm^−1^, the skeletal vibrations found in the region (here defined as <500 cm^−1^ instead of the previously reported <800 cm^−1^ by Xu et al. [22]), the symmetric stretching modes of C–C and C–O bonds (950–1200 cm^−1^), and the C–H deformation bands (1200–1500 cm^−1^) [80]. Gluten-specific bands are also identifiable in the wheat flour spectra but are absent from the starch spectrum. These include bands at 1003 cm^−1^, 1392–1454 cm^−1^, and 1657 cm^−1^, corresponding to aromatic ring vibrations (likely from tyrosine and phenylalanine residues), C–H deformation modes, and the amide I band (C=O stretching), respectively [30]. Importantly, Kizil and Irudayaraj [13] confirmed that the amide I region does not experience interference from carbohydrate-associated bands, allowing for selective analysis of protein components (see Figure 3). In addition to the amide I region, the disulfide stretching region (450–500 cm^−1^) is of particular interest for gluten protein analysis. Since this overlaps with the starch band, a correction strategy is implemented in several studies [50,51,65]. Herein, the interfering starch band is subtracted from the composite spectrum prior to deconvolution of the disulfide region. However, such a correction does not account for potential interaction effects between starch and gluten. 

At the wheat flour level, both Piccinini et al. [81] and Carcione et al. [80] investigated *Triticum aestivum* (soft wheat) and *Triticum durum* (durum wheat), with particular attention paid to the previously described spectral regions associated with starch. Although *T. durum* is less commonly utilized in the bakery sector, it was nonetheless included by Piccinini et al. [81] for the production of semolina (i.e., the milled fraction of *T. durum*) bread. Their findings revealed strong spectral similarities between the two flour types. Carcione et al. [80] conducted a more detailed comparative analysis of the spectral profiles by examining the intensity at 478 cm^−1^ relative to the C–O–C stretching band at 860 cm^−1^. The resulting intensity ratios were 1.8, 1.8, 1.9, and 2.2 for soft wheat, durum wheat, integrated soft wheat, and biological soft wheat, respectively. These minor differences indicate minimal variation between the wheat types, with the highest ratio observed in biological soft wheat, suggesting a remarkable molecular ordering.

The studies by Huen et al. [82] and Wang et al. [52] took into account frozen dough. The first study one used the bands located between 460–500 cm^−1^, 740–766 cm^−1^, 1645–1690 cm^−1^ (part of amide I region), and 3080–3200 cm^−1^ (part of OH stretching region) to visually distinguish between starch, yeast, gluten, and ice, respectively. These spectral measurements were performed on the inner dough part (~250 mg), sampled onto a microscope slide, covered and subsequently frozen. The spatial distribution was visualized through band integration of the previously mentioned bands, since they show high intensities in individual component spectra, and through a multiple regression approach, using full single-component spectra. These two mapping approaches enabled detailed visualization of the starch granule morphology, revealing both large (20–25 µm) and small (2–5 µm) granules, surrounded by gluten as fibrils, and liquid water present at dough spaces with no other components. However, the authors cautioned that attribution of the amide I region to gluten is not entirely definitive due to the variability introduced by protein secondary and tertiary structures. Additionally, the spectral region 740–760 cm^−1^, linked to added yeast, did not permit accurate mapping of that component. Instead of visualizing the presence of water in the frozen dough, the study by Wang et al. [52] focused on the ice-crystal-induced damage on the gluten network. Therefore, focus was placed on four common Raman parameters and the OH stretching band throughout traditional and ultra-sound-assisted freezing, monitored every five minutes by means of a fiber optic probe placed within the ultrasonic working chamber acquiring surface Raman spectra. Damage was observed by the overall decrease in the g–g–g disulfide conformation and a simultaneous increase in t–g–g conformation, alongside a decrease in intensity of the 760 cm^−1^ band and an increase in the I_850_/I_830_ tyrosine doublet ratio with prolonged freezing duration for both freezing methods. Analysis of the amide I region revealed a gradual reduction in α-helix and β-turns structures, accompanied by an increase in random coils and β-sheets. Furthermore, the peak position within the OH stretching band gradually decreased. This indicates damage to the gluten network caused by ice crystal formation, leading to the breakdown and weakening of stable disulfide bonds and hydrogen bonding. This damage appears to be less pronounced when ultrasound is applied, likely due to the more uniform formation of ice crystals and the cavitation effect, in comparison to conventional freezing. Another dough-level study, by Lancelot et al. [50], focused on the importance of the gluten structure during dough development, considering under-, optimal, and over-kneaded dough, as well as the presence of tris(2-carboxyethyl)phosphine (TCEP), a disulfide bond-reducing agent. The analysis was performed on dough pieces of ~2 g sampled onto a microscope slide and targeted characteristic gluten-associated spectral regions, notably the amide I and III bands, the disulfide stretching region, and tyrosine- and tryptophan-related markers. Disulfide bond analysis showed that the stable g–g–g conformation (~497 cm^−1^) increased with optimal kneading but decreased with overmixing in untreated dough. In contrast, TCEP-treated dough showed low g–g–g intensity and a predominance of the less stable t–g–g form throughout kneading. Up to optimal kneading, there was an increase in α-helices, the dominant secondary structure, and antiparallel β-sheets, while random coils and β-turns decreased. TCEP treatment disrupted this organization, suppressing the formation of both α-helices and antiparallel β-sheets. The tyrosine ratio remained consistently above one, even with TCEP, indicating stable hydrogen bonding. Tryptophan burial peaked at optimal kneading and decreased with overmixing, irrespective of the presence of TCEP. The secondary gluten structure based on the amide I region was also analyzed in lyophilized and pulverized dough by Correa et al. [49] to investigate the influence of salt (NaCl) or dietary fibers (modified celluloses and pectins) and the combination of both. The addition of salt or dietary fibers solely led to fewer α-helices and an increase in β-sheets (parallel and antiparallel). When both salt and hydrocolloids were incorporated simultaneously, a more pronounced decrease in α-helices was observed, alongside a greater overall proportion of β-sheets. These results are indicative of polypeptide unfolding and this facilitates crosslinking and aggregation of proteins. The influence of adding highland barley, so as to improve nutritional aspects, to freeze-dried and ground cake batter was evaluated by Wu et al. [83]. Raman analysis revealed that the disulfide bond region shifted toward higher wavenumbers, and along with the increased content of β-sheets and random coils, this indicates a decrease in structural stability upon addition. Additionally, a position shift occurs within the C–H stretching region near 2900 cm^−1^, which suggests competition between barley flour and proteins for hydrogen-bonding sites on the hydroxyl groups of water molecules. These findings aligned with thermomechanical properties: supplementation increased the water absorption, stabilization time, and protein weakening. However, the longer stabilization reflected reduced dough consistency rather than stronger gluten. The starch gelatinization and retrogradation parameters decreased, likely due to barley’s high water absorption. Baked cakes exhibited increased hardness and reduced adhesiveness and springiness, consistent with the disruptive effect of barley flour, producing a dense, less elastic structure. At the level of baked products, Raman spectroscopy has been applied from different perspectives, as seen in the studies by Sivam et al. [30] and Rodriguez and Kurouski [84]. The former investigated the influence of polyphenols and pectins in a bread formulation on conformational changes, particularly those of gluten. Differences between control and treated breads, which were all lyophilized and pulverized, could be observed at specific bands, despite the overall spectral similarities. For example, the band at 1003 cm^−1^, which is present in gluten, flour, and control bread but absent in starch and fortified samples, indicates interactions between the additives and gluten. A pronounced band at 1656 cm^−1^ is observed in the gluten spectrum but appears less intense in treated formulations, which can be interpreted as α-helices interacting with the added components. In contrast, Rodriguez and Kurouski [84] used Raman spectroscopy for the quantification of macronutrients in various final products, including bread and crackers by means of spectra acquisition of the surface. Consistent with earlier findings, the amide I band (~1656 cm^−1^) functioned as a protein marker of which the intensity varied with protein content, while the intensities of carbohydrate-related bands, specifically vibrations associated with C–O–C and C–O–H in the 400–1400 cm^−1^ region, varied with the carbohydrate content. Two other studies investigated final products, though, focusing on starch retrogradation in the light of bread staling [81,85]. The previously mentioned study by Piccinini et al. [81] on semolina bread used 2D-COS analysis to monitor the changes over a 20-day storage period measured on the sampled inner part of the bread crumb. The main Raman band at 480 cm^−1^ exhibited a narrower FWHM and a position shift throughout continued storage, which aligns with earlier studies on isolated wheat starch and is interpreted as increased molecular ordering. This is in accordance with the increasing crumb hardness measured through texture analysis. Furthermore, the intensity of the 850 cm^−1^ shoulder decreased, while a new band appeared at 765 cm^−1^. The latter is attributed to CH_2_ rocking and is possibly linked to amylopectin, given the presence of α-1,6-glycosidic linkages in its branched structure, which involve CH_2_OH groups. An et al. [85] focused on detecting staling in Chinese steamed bread, aiming to quantify staling indicators using spectral data fusion from near-infrared (NIR), mid-infrared (MIR), and Raman spectroscopy. Spectral analyses were performed on lyophilized, ground and sieved samples. The study concluded that models based solely on Raman data outperformed those based on NIR and MIR alone in predicting three key indicators of staling: soluble starch amylose content, relative crystallinity, and hardness. However, combining all three techniques yielded a higher prediction accuracy.

## 7. Conclusions and Future Perspectives

Given its numerous advantages, including its ability to perform rapid, non-destructive, in situ measurements without the need for sample preparation, and under these conditions allowing simultaneous identification of the present components and their molecular states, Raman spectroscopy is a promising tool for broader implementation in the food industry. As highlighted by Boyaci et al. [86], a comprehensive understanding of the food matrix composition, as well as how this is impacted by environmental factors and processing conditions, which significantly contribute to the final product properties, is crucial. In this context, the ability to monitor dynamic changes becomes increasingly important and Raman spectroscopy, owing to its molecular-level sensitivity and versatility, is well-suited to support such monitoring efforts. 

Focusing specifically on the bakery sector, in which wheat flour constitutes the principal ingredient, Raman spectroscopic data can provide insight into both starch and (gluten) proteins, as the most abundant and functionally relevant components. In the case of starch, particular attention is paid to the prominent Raman band near 480 cm^−1^, for which the FWHM is frequently analyzed as an indicator of changes in molecular ordering, such as those induced by gelatinization and retrogradation processes. For gluten proteins, four recurring spectroscopic parameters are of particular interest to deduce changes related to processing and added ingredients such as dietary-fiber-related compounds. These include the disulfide bond region, characterized by distinct conformations: g–g–g, t–g–g, and t–g–t, located within 500–560 cm^−1^; the aromatic amino acid bands associated with tryptophan (760 cm^−1^) and tyrosine (expressed as a ratio between bands at 850 and 830 cm^−1^), which reflect changes in the local microenvironment of these residues; and the amide I region, which provides information on secondary protein structures.

However, to fully harness the potential of Raman spectroscopy for food applications, and particularly within the context of bakery products, further research is needed under realistic processing conditions. Future studies should extend beyond simplified or isolated systems, which have dominated much of the existing literature, and instead focus on complex food matrices, such as wheat flour doughs and their final baked products, in which the simultaneous presence of multiple components adds interpretative complexity to spectral data. Even to date, those studies on complex matrices have remained overly focused on the characterization of single components. This highlights the need to develop appropriate spectral processing to resolve spectral overlap accurately. Another hurdle to the implementation of Raman spectroscopy as a PAT tool in bakery manufacturing is the lack of insights into the influence of and the need for sampling since treatments such as lyophilization and pulverizing are incompatible with real-time monitoring. From a mapping perspective, limited effort has also been directed toward optimizing microscopic parameters, including the grid size, spot size, acquisition time, and selection of spectral markers to differentiate components. Addressing these challenges is essential to enable visualization of food constituents and their structural transformations during processing or in response to ingredient modifications. Such progress will be critical to translate laboratory-scale insights into practical, industrially relevant applications. 

## Figures and Tables

**Table 1:** Band assignments for Raman spectral data of wheat starch.

Raman (cm^−1^)	Band Assignment ^1^	Reference
411, 443, 480, 530, 576, 618	ν(C–C–O), δ(C–C–O) pyranose ring skeletal modes	[29]
721, 770, 779	ν(C–C) ring mode	[13,29]
840; 848–868	C-1–H, CH_2_ deformation, X–C-1–H group stretching and bending (with X = C-2, O-1, or O-5)	[13,30]
865, 868	ν_s_(C-1–O–C-5), δ(CH_2_), δ(C–H)	[29,30]
910 (shoulder)	δ(C–OH), δ(CH_2_), δ(C-1–H)	[29]
942	CH_2_ vibrations	[30]
943	ν_s_(C-1–O–C-4′)	[29]
941–944	X–C-1–H group stretching and bending (with X = C-2, O-1, or O-5)	[30]
1003	CH_2_-related mode	[29]
1053	ν(C–OH), δ(C–OH) modes, ν(C–C)	[29]
1083	C–O group, X–C-1–H group stretching and bending (with X = C-2, O-1, or O-5)	[30]
1076, 1084	δ(C–OH) bending	[13,29]
1110 (shoulder)	C–C, C–O, C–H-related mode	[29]
1127	ν(C–OH), δ(C–OH), ν(C–O)	[29]
1155 (shoulder)	ν_a_(C-1–O–C-4′) α-1,4-glycosidic linkage	[29]
1205	δ(C–H)	[29]
1255–1263	C-1–O–H and/or C-6–O–H vibrations, CH_2_OH vibrations	[30]
1264, 1272	δ(CH_2_), C–OH, CH_2_OH (side chain)-related mode	[13,29]
1305	δ(C–H)	[29]
1331–1339	C–H and CH_2_-related modes	[30]
1335	C–O–H bend, CH_2_ twist	[13]
1341	δ(CH_2_) twisting, δ(C–OH) bending	[29]
1350	CO stretching, COH bending	[30]
1381; 1370–1410	δ(C–OH), δ(C–H) bending, δ(CH_2_) scissoring	[13,29]
1403 (shoulder)	δ(C–H) bending	[29]
1460, 1462	δ_s_(CH_2_) twisting, C–H bending	[13,29,30]
1355–1599	C–H and CH_2_-related modes	[30]
2910; 2800–3000	ν(C–H)	[13,22,28,29]
3000–3600	ν(O–H)	[13,22,28]

^1^ δ: mode involves a change of bond angle (deformation or bending); ν: mode involves a change of bond length (stretching). Subscript ‘s’: symmetrical; subscript ‘a’: asymmetrical.

**Table 5:** Overall structural changes (increases ↑ or decreases ↓) of gluten or gliadin proteins when supplemented with phenolic components (phenolic acids or flavonoids/glycosides) or dietary fiber (DF)-related compounds compared to control untreated samples.

Sample Type	Disulfide Region (%)	Tryptophan (Intensity)	Tyrosine Doublet Ratio	Amide I ^1^ (%)	Reference
**Washed-out gluten/phenolic acids**	↑ t–g–g, ↓ g–g–g	Mostly insignificantly changed	↑	↑ AGR, pβS and βS, ↓ αH and βT	[51]
**Washed-out gluten/phenolic acids**	↑ t–g–t and t–g–g, ↓ g–g–g	Mostly insignificantly changed	Mostly insignificantly changed	↑ AGR, pβS and hβS, ↓ αH and βT	[53]
**Washed-out gluten/phenolic acids**	↓ or insignificantly changed g–g–g, ↑ and ↓ t–g–g, and mostly insignificantly changed t–g–t	↑	Mostly insignificantly changed	Measured with FTIR	[63]
**Washed-out gluten/phenolic acids**	↑ t–g–t and t–g–g, ↓ g–g–g	↑	Mostly insignificantly changed	↑ pβS and hβS, ↓ αH and aβS	[64]
**Washed-out gluten/phenolic acids**	Mostly insignificantly changed	Mostly insignificantly changed (exception: ↓ for highest concentration of SYN ^2^)	↓	↑ AGR, ↓ βS and βT	[65]
**Gliadin/salt/salt + Q ^3^**	↑ t–g–t and t–g–g, ↓ g–g–g	↓ caused by salt, ↑ or insignificantly changed by salt + Q	↑ caused by salt, ↓ or insignificantly changed by salt + Q	Measured by FTIR	[57]
**Gliadin/anthocyanins**	↑ t–g–g, ↓ g–g–g	N.S. ^4^	↓	↑ βS, ↓ βT	[62]
**Washed-out gluten/flavonoids/glycosides**	↑ t–g–g, ↓ t–g–t	↑ or insignificantly changed	↓ or insignificantly changed	↓ AGR, αH, βT and aβS caused by flavonoids, ↑ AGR, pβS, and βS, ↓ βT and aβS caused by glycosides	[66]
**Gliadin/flavonoids/glycosides**	↑ g–g–g, ↓ t–g–t and t–g–g	Mostly insignificantly changed	Mostly insignificantly changed	↑ αH, HbaβS, HbβT and aβS, ↓ AGR and pβS	[67]
**Gluten/WEAX ^5^**	↑ g–g–g and t–g–g, ↓ t–g–t at 25 °C, ↑ and ↓ (very compound, concentration and temperature dependent)	↓ <50 °C, ↑ >70 °C	↓ at different temperatures (25–95 °C)	Measured with FTIR	[42]
**Washed-out gluten/DF**	↑ t–g–t, ↓ g–g–g	↑ and ↓ (very compound dependent)	Slight changes	↑ aβS, ↓ pβS	[46]
**Gluten/WEAX**	↓ t–g–t	↑ at 25 °C, slight changes > 55 °C	↑ and ↓ (temperature dependent)	Measured with FTIR	[47]
**Washed-out gluten/hydrocolloids**	↑ t–g–t and t–g–g, ↓ g–g–g	↑ for LBG ^12^, ↓ for XG ^13^, GG ^14^ and P ^15^	↑ for P and LBG, ↓ for XG and GG	↑ αH, solvated helix, and βS, ↓ aβS and βT for LBG, ↑ aβS, βS, βT, solvated helix, and RC, ↓ αH for XG, GG and P	[68]
**Washed-out gluten/DF**	↑ t–g–t and t–g–g, ↓ g–g–g	N.S.	↓ (exception: ↑ for oat fiber)	↑ αH and pβS, ↓ βS and βT	[69]
**Washed-out gluten/DF**	↑ t–g–t, ↓ g–g–g (exception: ↑ g–g–g caused by CRB ^6^ and FLX ^7^)	↑	↓	↑ and ↓ (very compound and concentration dependent)	[70]
**Washed-out gluten/DF**	↑ and ↓ (very compound and concentration dependent)	↑ and ↓ (very compound and concentration dependent, however mostly increasing intensity with increasing concentration)	↓	↑ aβS, ↓ αH	[71]
**Washed-out gluten/DF**	↑ g–g–g (exception: for ↓ IN), ↑ and ↓ t–g–t and t–g–g (very compound and concentration dependent)	↓ (exception: ↑ for highest concentration of MCC and CP ^10^)	↓ for MCC and IN, ↑ for AP ^11^ and CP	↑ aβS, HbaβS, AGR (in case of high contents of pectin), ↓ αH, pβS, βS, HbβT	[72]
**Washed-out gluten/DF**	↑ g–g–g (exception: ↓ for IN ^8^), ↓ or absence of t–g–t (except for MCC ^9^)	↑	↓	↑ AGR, HbaβS, and βT, ↓ αH, pβS, βS, hβS and HbβT	[73]
**Washed-out gluten/moisturized DF/non-moisturized DF**	↑ t–g–t, ↓ g–g–g	↓	Mostly insignificantly changed	Measured with FTIR	[74]
**Washed-out gluten/oil pomaces**	↑ t–g–t and t–g–g, ↓ g–g–g	↓	Mostly insignificantly changed	Measured with FTIR	[75]
**Gluten/wheat bran DF**	N.S.	↑	↓	Measured with FTIR	[76]
**Washed-out gluten/KGM ^16^**	↑ t–g–t and t–g–g, ↓ g–g–g (except for 81% hydration, g-g-g remains dominant structure)	N.S.	↑ for 81% hydration, ↓ 55% hydration	↑ βS, ↓ αH	[77]

^1^ Amide I band abbreviations: AGR—aggregate, RC—random coil, hβS—hydrated β-sheet, pβS—pseudo-β-sheet, βS—β-sheet, aβS—antiparallel-β-sheet, HbaβS—hydrogen-bonded antiparallel-β-sheet, βT—β-turn, HbβT—hydrogen-bonded β-turn, αH—α-helix. ^2^ Sinapic acid. ^3^ Quercetin. ^4^ Not specified. ^5^ Water-extractable arabinoxylan. ^6^ Cranberry. ^7^ Flax. ^8^ Inulin. ^9^ Microcrystalline cellulose. ^10^ Citrus pectin. ^11^ Apple pectin. ^12^ Locust bean gum. ^13^ Xanthan gum. ^14^ Guar gum. ^15^ Pectin. ^16^ Konjac glucomannan.

**Table 6:** Methodological setup of research conducted on complex matrices, as outlined in the corresponding article.

Sample Type	Raman Device	Laser Wavelength (nm) and Power (mW)	Resolution (cm^−1^)	Number of Scans/Accumulations	Raman Shift Range (cm^−1^)	(Pre-)Processing Steps	Reference
**Bread (with/without polyphenols and pectins)**	LCM-Raman ^1^	785 5	N.S. ^2^	40	200–3200	N.S.	[30]
**Dough (with/without salt, celluloses, and pectins)**	FT-Raman	1064 500	6	1000	N.S.	Baseline correction, normalization, deconvolution	[49]
**Dough (with/without TCEP ^3^** **)**	Raman microscope	785 40	9	12	400–1800	Calibration, baseline correction, normalization, deconvolution	[50]
**Frozen dough**	Micro-miniature Raman spectrometer	785 N.S.	3	N.S.	175–4000	Baseline calibration, deconvolution	[52]
**Wheat flour (soft, hard)**	LCM-Raman	785 50	N.S.	N.S.	N.S.	Baseline correction	[80]
**Semolina bread crumb**	FT-Raman	1064 N.S.	4	128	250–3500	Blackmann–Harris 3-term apodization, baseline correction, normalization, zero filling	[81]
**Frozen bread dough**	Confocal Raman (imaging protocol)	N.S. N.S.	200 × 200	40,000	N.S.	N.S.	[82]
**Cake batter**	N.S.	633 10	N.S.	3	100–4000	N.S.	[83]
**Baked products (e.g., bread, crackers)**	Handheld Raman	830 495	N.S.	20–25	N.S.	Baseline correction, normalization, smoothing	[84]
**Chinese steamed bread**	Portable Raman	785 300,000	N.S.	N.S.	200–2000	Smoothing, standard normal variate transformation	[85]

^1^ Laser confocal micro-Raman (employed for single-point measurements). ^2^ Not specified. ^3^ Tris(2-carboxyethyl)phosphine.

## Data Availability

Data available upon reasonable request to the corresponding author.

## Abbreviations

The following abbreviations are used in this manuscript:

CCD	Charge-coupled device
PAT	Process Analytical Technology
VIS	Visual light
UV	Ultra-violet
NIR	Near-infrared
FT-Raman	Fourier-transform Raman
RPO	Rapeseed oil
CRW	Carnauba wax
XRD	X-ray diffraction
SAXS	Small-angle X-ray scattering
DSC	Differential scanning calorimetry
FTIR	Fourier transform infrared spectroscopy
NMR	Nuclear magnetic resonance
T_o_	Onset temperature
T_p_	Peak temperature
T_c_	Conclusion temperature
FWHM	Full width at half maximum
NWS	Normal wheat starch
WWS	Waxy wheat starch
2D-COS	Two-dimensional correlation spectroscopy
LCM-Raman	Laser confocal micro-Raman
N.S.	Not specified
HMW	High molecular weight
LMW	Low molecular weight
-SH	Thiol
S-S	Disulfide bonds
SE-HPLC	Size-exclusion high-performance liquid chromatography
RP-HPLC	Reversed-phase high-performance liquid chromatography
LC-MS	Liquid chromatography–mass spectrometry
CD	Circular dichroism
IR	Infra-red
g–g–g or SSg–g–g	Gauche–gauche–gauche
g–g–t or SSg–g–t	Gauche–gauche–trans
t–g–g	Trans–gauche–gauche
t–g–t or SSt–g–t	Trans–gauche–trans
AGR	Aggregate
pβS	pseudo-β-sheet
βS	β-sheet
αH	α-helix
βT	β-turn
aβS	Antiparallel-β-sheet
Trp	Tryptophan
Tyr	Tyrosine
Phe	Phenylalanine
RC	Random coil
hβS	Hydrated β-sheet
HbβT	Hydrogen-bonded β-turn
HbaβS	Hydrogen-bonded antiparallel β-sheet
SSL	Sodium stearoyl lactylate
DATEM	Diacetyl tartaric acid esters of monoglycerides
WEAX	Water-extractable arabinoxylan
Q	Quercetin
KGM	Konjac glucomannan
DF	Dietary fiber
SYN	Sinapic acid
CRB	Cranberry
FLX	Flax
IN	Inulin
MCC	Microcrystalline cellulose
CP	Citrus pectin
AP	Apple pectin
LBG	Locust bean gum
XG	Xanthan gum
GG	Guar gum
P	Pectin
TCEP	Tris(2-carboxyethyl)phosphine
MIR	Mid-infrared

## Appendix A

### Appendix A.1. Materials and Methods

This Materials and Methods section covers the samples that were Raman spectrally measured to support the insights in this review of wheat starch and gluten, in an isolated state and within more complex matrices, and of which the illustrative spectra can be found in Figure 3. Additionally, the preparation and measuring setup of the illustrative Raman mapping used in Figure 2B is also included.

#### Appendix A.1.1. Materials

The materials included, on the one hand, commercial native wheat starch, native wheat gluten, and wheat flour, and on the other hand, 10% carnauba wax (CRW) added to rapeseed oil (RPO) oleogel.

#### Appendix A.1.2. Dough-Making Procedure

Dough was prepared using a 50 g Brabender Farinograph (Brabender GmbH & Co. KG, Duisburg, Germany). A sample was prepared by mixing 49.65 g of *Epi B* wheat flour with 28.8 g of distilled water, with the latter determined based on the water absorption required to reach a dough consistency of 500 BU, as defined by the Farinograph analysis. Mixing was conducted at 63 rpm (standard operating speed) for 7.2 min, corresponding to the stability time derived from Farinograph testing. To prevent dehydration prior to Raman analysis, the dough was stored in a sealed plastic container. Sampling was performed from the core of the dough mass to minimize the surface drying effects and analysis was performed within one hour post-mixing.

#### Appendix A.1.3. Raman Measurement and Data Processing

Raman spectral measurements were performed on all the sample types using a Bruker Senterra II Raman microscope (Bruker Optics GmbH & Co. KG, Ettlingen, Germany), equipped with a 785 nm excitation laser operating at 100 mW. Samples were placed on microscope slides and measured without additional preparation. Spectra were acquired in the range of 80–3500 cm^−1^, with a spectral resolution of 3–5 cm^−1^. For each sample, three accumulations of 30 s single-point measurements were collected using a 20× objective and a 50 µm aperture. All the spectra from the different sample types were only scaled using an in-house developed MATLAB (R2025a) script and presented in the relevant Raman shift ranges.

Raman mapping of the RPO oleogel containing 10% CRW at 20 °C was conducted using the same Bruker Senterra II Raman microscope, with a 785 nm excitation laser. Measurements were taken at each of the 52 × 52 points, with three repetitions per point and a single acquisition time of 15 s, covering an area of 52 × 52 µm. The visualization in terms of the intensity ratio was achieved by considering the bands 1605 cm^−1^ and 1663 cm^−1^ for CRW and RPO, respectively.
